# Nanotechnology-Based Vaccines for Allergen-Specific Immunotherapy: Potentials and Challenges of Conventional and Novel Adjuvants under Research

**DOI:** 10.3390/vaccines8020237

**Published:** 2020-05-20

**Authors:** Litty Johnson, Albert Duschl, Martin Himly

**Affiliations:** Department of Biosciences, University of Salzburg, 5020 Salzburg, Austria; litty.johnson@sbg.ac.at (L.J.); albert.duschl@sbg.ac.at (A.D.)

**Keywords:** AIT, allergy, alum, calcium phosphate, microcrystalline tyrosine, CpG oligonucleotide, nanoparticles

## Abstract

The increasing prevalence of allergic diseases demands efficient therapeutic strategies for their mitigation. Allergen-specific immunotherapy (AIT) is the only causal rather than symptomatic treatment method available for allergy. Currently, AIT is being administered using immune response modifiers or adjuvants. Adjuvants aid in the induction of a vigorous and long-lasting immune response, thereby improving the efficiency of AIT. The successful development of a novel adjuvant requires a thorough understanding of the conventional and novel adjuvants under development. Thus, this review discusses the potentials and challenges of these adjuvants and their mechanism of action. Vaccine development based on nanoparticles is a promising strategy for AIT, due to their inherent physicochemical properties, along with their ease of production and ability to stimulate innate immunity. Although nanoparticles have provided promising results as an adjuvant for AIT in in vivo studies, a deeper insight into the interaction of nanoparticle–allergen complexes with the immune system is necessary. This review focuses on the methods of harnessing the adjuvant effect of nanoparticles by detailing the molecular mechanisms underlying the immune response, which includes allergen uptake, processing, presentation, and induction of T cell differentiation.

## 1. Introduction

Atopic diseases are a major health problem due to their increased prevalence in developed nations [1]. According to an estimate of the European Academy of Allergy and Clinical Immunology (EAACI) in 2016, around 150 million European citizens suffer from allergy and it is predicted that in the foreseeable future more than half of the population, especially in developed countries, will be afflicted by allergy [2]. This might be due to the substantial increase in allergy triggers. It has been proposed that the aggravation of allergic disease can be contributed by factors like industrialization, urbanization, environmental changes, lifestyle, and diet changes [3,4]. The adoption of a Western lifestyle, including its influence on exposure to microbiota (excessive hygiene) in the development of allergic diseases have been suggested to be one of the reasons [5]. However, there are no simple explanations for mechanisms underlying the rising incidence of allergies. These disorders can impair the quality of life of people and contribute to a decreased socioeconomic growth [6]. This compels the need to develop effective treatment strategies.

The current treatment methods involve the use of anti-symptomatic drugs like anti-histamines, corticosteroids, anti-leukotrienes (providing symptomatic relief), and allergen-specific immunotherapy (AIT). AIT is the only available curative treatment approach for allergy [7]. The global allergy treatment market for anti-symptomatic treatment of allergy is about 89% and it is expected to continue its dominance over the next years. Even though the AIT segment shows the highest compound annual growth rate (CAGR) of 8.5%, the overall market share of AIT is expected to stay low [8]. This is due to poor patient compliance, uncertain success, lack of awareness, adverse effects, and long duration of treatment [9]. Thus, there is an urge for concerted efforts to develop novel, safe, and effective treatment strategies for allergies without compromising patient safety. This review focuses on the potentials and limitations of conventional and novel adjuvants with a special focus on the suitability of nanoparticulate carriers for improved AIT vaccine strategies.

## 2. Allergen-Specific Immunotherapy—The Principle and Novel Approaches

**Figure 1:**
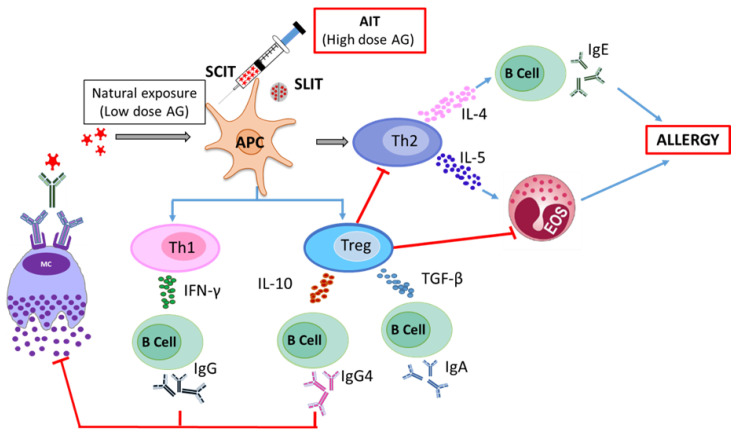
Mechanisms of allergy induction and allergen-specific immunotherapy.

The design of an effective therapeutic strategy requires good insight into the mechanistic development of allergic diseases and the standard treatment methods. The mechanism of development of an allergic response can be explained by two stages, a sensitization phase and an effector phase. In the sensitization phase, an allergen is encountered by antigen-presenting cells (APCs) followed by its uptake, antigen processing, and presentation, leading to the activation of immune cells. This activation further results in the polarization of the T helper (Th) cell to the Th2 phenotype, which stimulates the B cells to produce allergen-specific immunoglobulin E (IgE) antibodies. The allergen-specific antibodies can bind to high-affinity IgE receptors (FcεRI) and prepare the immune effector cells such as mast cells and basophils for a subsequent allergen-driven effector function. Upon re-confrontation with the same allergen, the allergens bind to the specific IgE antibody on mast cells, leading to degranulation and release of pre-formed mediators, causing immediate hypersensitivity reactions. The release of inflammatory mediators by mast cells also changes the cytokine milieu, which promotes the further development of allergic late-phase symptoms (Figure 1) [10].

Allergen-specific immunotherapy (AIT) aims to induce T cell tolerance by the administration of relatively high doses of allergen. In contrast to the differentiation of naive T cells to Th2 cells in the course of allergic sensitization, the T cell differentiation during successful AIT is either directed towards a Th1- or regulatory T cell (Treg)-driven response in AIT. This intended immune modulation produces cytokines like interleukin 10 (IL-10), transforming growth factor-β (TGF-β), and interferon-γ (IFN-γ), which interact with B cells to produce allergen-specific IgG4 and IgA antibodies that help to protect patients against further allergic responses. The activation of Treg cells suppresses Th1 and Th2 cell recruitment, attenuates Th2 immune responses and, in the long run, downregulates allergen-specific IgE. These processes together finally result in the inhibition of mast cell degranulation, and lowering or abolishing the symptoms of allergy (Figure 1) [11].

The first application of AIT dates back to more than 100 years ago [12]. Despite its effectiveness, the application of conventional AIT bears the risk of adverse effects that reduce patient compliance [13]. The development of novel AIT vaccine approaches should address these limitations along with the improved induction of long-term tolerance to allergens. Some of the strategies that have been proposed in therapy are described below.

### 2.1. Application of Molecular AIT Strategies

The conventional AIT strategies involve the use of allergenic extracts for the induction of immune tolerance. The variable quality of the extracts with seasonal or manufactural changes or even with the entire absence of some immunologically active substances, creates hurdles in their application [14,15]. Thus, molecular AIT approaches exhibit a huge potential in transforming AIT to the next level. Some of the prominent advancements in this regard include the production of highly purified and well-characterized recombinant allergens, design of peptide vaccines with allergen-derived T cell epitopes, and synthetic hypoallergenic variants. Recombinant allergens display the advantage of high quality and better reproducibility in the production process, thereby improving the safety and efficacy profile of AIT [16]. A two-year clinical study with subcutaneous administration of recombinant Bet v 1 showed better efficacy, compared to the conventional allergenic extracts. This recombinant vaccine also exhibited clinical relevance as it showed significant increase in allergen-specific IgG4 antibodies, along with reduction in skin sensitivity [17]. The design of peptides with T cell epitopes was considered to be another effective technique to reduce or even totally circumvent IgE binding, contributing to a low risk of anaphylaxis. However, the outcome of this preliminary study turned out to be clinically ineffective, as it failed to induce IgG antibodies, putatively due to the fact that the peptides used were too short [18]. Later, Spertini et al. developed longer peptides of Bet v 1 adjuvanted with alum to induce tolerance. This strategy was found to be clinically effective in phase 2 clinical trials [19]. Hypoallergenic derivatives of allergens have the property to bind significantly less or no IgE. They can still induce an allergen-specific IgG4 response and thus can compete with IgE binding, leading to a reduction in the immediate adverse effects [15]. A hypoallergenic fold variant of Bet v 1 has been successful in early clinical trials and has now reached the clinical phase 3 [20].

### 2.2. New Routes of Allergen Administration

A new route for AIT should reduce the current dosing regimens and associated risks. Epicutaneous and intralymphatic routes are some of the novel routes that hold great promise for AIT. Epicutaneous immunotherapy utilizing a tape-stripping approach displayed better clinical efficiency and improved patient compliance. Notably, this treatment option was much less invasive and could be administered by the patients themselves [21]. Similar in effectiveness, a randomized clinical trial with intralymphatic administration of grass pollen allergen showed an equivalent but long-lasting tolerance when compared to subcutaneous immunotherapy, reducing the overall treatment time [22]. Using other routes of administration such as oral immunotherapy will become more routine in the future, especially for the treatment of food allergy [23].

### 2.3. Fusion of Allergens with Immune Response Modifiers or Adjuvants

The objective of using hypoallergenic variants of intact allergen or of allergen-derived peptides in AIT is to reduce the unwanted side effects associated with conventional AIT. However, in some cases it lowers immunogenicity of the active substance as the IgE epitopes are modified, which leads to a lower uptake by the antigen-processing cells [24]. The combination of the adjuvants or immune response modifiers with the allergen as a single moiety could tackle this complication with decreased detrimental effects. Coupling of allergens to modifiers of the innate immune response has been shown to inhibit mast cell and basophil degranulation, while preserving the immunogenicity of the active ingredient [25]. The fusion of the major cat allergen Fel d 1 to a cell-penetrating peptide derived from the translocation sequence of mice modular antigen transporter (MAT) and a part of the invariant chain has been reported to be safe and effective in a first clinical study [22]. Allergen–nanoparticle coupling is considered to be a promising strategy to improve AIT. Andersson et al. demonstrated the potential of recombinant Fel d 1 coupled to carbohydrate particles (CBPs) through covalent bonding, as an effective tool in AIT. The sepharose-based CBPs of 2 µm size were coupled with Fel d 1, in the presence of phosphate buffered saline. The authors investigated the potential of the particulate adjuvant in a mouse model for cat allergen and found a significant increase in the IgG-to-IgE ratio concomitant, with decreased airway hyper-reactivity and infiltration of eosinophils. Hence, they proposed that allergen-complexed particles exhibit the potential to improve allergy treatment [26].

## 3. Classical Adjuvants in AIT—Mechanistic Insight

Adjuvants, in general, are pharmaceutical aids that are incorporated into vaccine preparations, to improve the desired immune response for a therapy intended to prevent or ameliorate the state of a disease [27]. The main objective for incorporation of adjuvants in AIT is to increase the efficacy and safety of the treatment, moreover, they are expected to enhance and simplify immunization regimens. They act through various mechanisms such as by the formation of a depot at the site of injection, increasing the capture by APC, and modulation of innate immunity [28]. In this section, a brief overview on the mechanism, safety, and efficacy of the conventional and novel adjuvants are discussed.

### 3.1. AIT Adjuvants in Clinical Practice

Alum, calcium phosphate, microcrystalline tyrosine (MCT), and monophosphoryl lipid A (MPL) are the conventional adjuvants used in AIT and these represent the only adjuvants that can be currently found in marketed AIT products. All these, with the exemption of MPL are considered as particulate systems that establish a depot effect at the administration site. This physical property makes them suitable for sustained release of allergens in AIT.

#### 3.1.1. Alum

**Figure 2:**
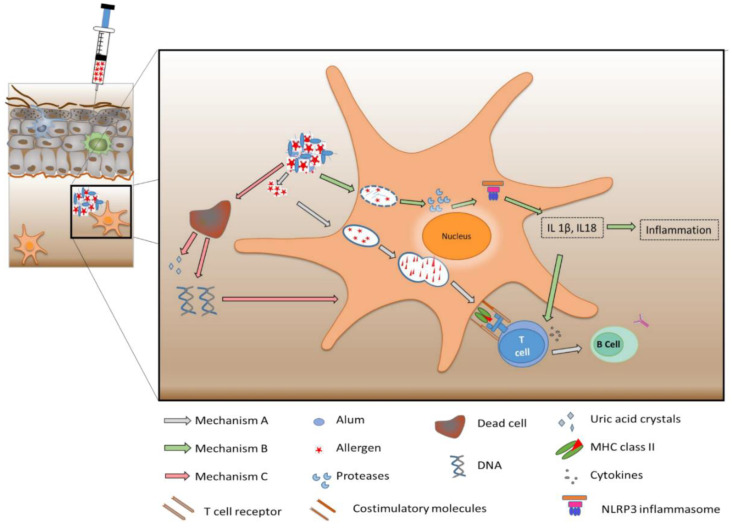
Scheme of three mechanisms of alum’s action as adjuvant for allergen-specific immunotherapy (AIT). Mechanism A—depot formation leading to immunomodulation; Mechanism B—NLRP3 inflammasome activation resulting in allergen-specific antibody production; and Mechanism C—induction of self-DNA release promoting dendritic cell maturation.

Alum is the most prominent adjuvant with a long history of use in AIT. The wide applicability of alum might be associated with its long history, ease of preparation, and good stability [29]. Initially, alum was considered to exhibit immunomodulatory properties only involving the process of depot formation. Alum adsorbs antigens onto its surface, mostly driven by electrostatic interactions (with its hydroxyl groups) at pH values slightly below the isoelectric points of the proteins to be adsorbed [30]. Due to its low solubility, larger agglomerates of the particulate matter in the micrometer size range are formed in the tissue and in the local lymphatic organs [31,32]. Here, the adsorbed antigens are released over a longer period through rapid chelation with alpha-hydroxycarboxylic acid in the interstitial fluid [33,34]. The released antigens are further engulfed by APCs, taken up into the cells, proteolytically processed and presented for initiating a potent immune response (Figure 2, Mechanism A). However, recent studies have challenged the central role of depot formation and actually proved its insignificance, as the removal of depot in the injection site at an early stage did not influence the antigen-specific T cell or B cell response [35]. Further investigations explored the propensity of alum to initiate inflammation. The endocytosis of the crystalline structures of alum can destabilize the endosome by inducing swelling and leakage of molecules like proteases and ions into the cytosol [36,37,38]. This drives NLRP3 (NOD-like receptor protein 3) inflammasome activation, which promotes the production of allergen-specific antibodies through pro-inflammatory cytokine release (Figure 2, Mechanism B) [37]. However, this hypothesis has also been conflicted by several contradictory reports. Preliminary in vivo studies reported that mice deficient in the NLRP3 gene showed a reduced antibody response [39]. In contrast, other reports displayed the insignificance of the NLRP3 gene deficiency in the production of allergen-specific antibodies [40,41]. Further investigations led to the discovery of a third mechanism, which involved the induction of self-DNA release by alum. A detailed analysis of the cytotoxic effects of alum detected increased concentrations of self-DNA entrapped in the alum nodules [42,43]. Experimental evidence has been provided that these nodules also contained significant amounts of myeloperoxidase and citrullinated histone, which might be an indication that ETosis (a cell death pathway involving the intentional release of cellular material) occurs at the site of the injection. The cytotoxicity of alum led to cell damage resulting in the release of uric acid and self-DNA [44,45]. The release of uric acid or self-DNA can act as a trigger for activation of immature dendritic cells (Figure 2, Mechanism C) [44]. The release of self-DNA could contribute to the adjuvant activity of alum through the activation of an IRF3-dependent and independent pathway. In the IRF3-independent pathway, the allergen-specific IgG antibodies are produced by an efficient T cell response (with T follicular helper cells) along with B helper cells activation. Whereas, in the IRF3-dependent pathway, antigen-loaded inflammatory monocytes could induce the production of IgE antibodies through the differentiation of T cells to the canonical T helper cells [43,46]. Even though the literature stipulate self-DNA induction as an important mechanism of alum’s adjuvant action, further evaluation is necessary to confirm this.

#### 3.1.2. Calcium Phosphate

Calcium phosphate is a rarely used adjuvant in AIT vaccines [47]. However, double blind placebo-controlled studies have demonstrated the effectiveness and safety of calcium phosphate for AIT [48]. The proposed mechanism of its immunomodulatory effect is the formation of a depot, as it readily adsorbs the antigen. Calcium phosphate exhibits less propensity to cause tissue irritation compared to alum. Moreover, studies with calcium phosphate nanoparticles induced a higher IgG titer and lower IgE response [49]. This suggests that calcium phosphate especially in its nanoform might act as an alternative adjuvant to alum.

#### 3.1.3. Microcrystalline Tyrosine

Microcrystalline tyrosine (MCT) serves as an adjuvant in AIT. It is an amino acid formulation that displays a high absorption capacity at a neutral pH. The short half-life of 48 h in the tissue (biodegradability) and its biocompatibility makes it a better adjuvant compared to alum [50,51]. Similar to alum and calcium phosphate, MCT forms a depot at the injection site and induces comparable Th1 stimulation. However, a lower IgE immune response was observed, which could lead to less adverse effects, compared to the former examples [52]. MCT is currently in use as an ultra-short course vaccine for seasonal allergic rhinitis [53].

#### 3.1.4. Monophosphoryl Lipid A (MPL)

MPL is derived from lipopolysaccharides (LPS) through the removal of a phosphate group and one ester-linked fatty acid chain from the reducing end of the lipid A disaccharide [54]. This structural modification preserves the immunomodulation potential of LPS without the induction of undesirable effects. The decreased toxic effects of MPL are correlated with the diminished expression of genes associated with the MyD88-dependant pathway, while its immunomodulatory property is maintained by the identical expression of genes associated with the TIR-domain-containing adapter-inducing interferon-β (TRIF)-dependent pathway [55]. Thus, MPL is considered as a toll-like receptor 4 (TLR4) agonist with a bias towards TRIF-associated signaling [56]. MPL as an adjuvant aids in initiating an immune response through the activation of APCs and induction of a Th1 cytokine cascade. Here, cytokines including IL-2 and IFN-γ are produced, leading to the stimulation of Th1 cells [57,58]. Moreover, it was reported to activate monocytes and macrophages, leading to elevated and faster phagocytosis, antigen processing, and presentation [59]. MPL by itself displays a poor bioavailability, due to its low solubility in water. Therefore, it is often used in combination with other adjuvants to increase efficacy [60]. Modified allergen tyrosine-adsorbed (MATA) monophosphoryl lipid A (MPL) immunotherapy formulations are commercially available for the treatment of grass, birch, and mugwort pollen allergies [61]. The synergetic adjuvant effect of MPL and MCT have been previously documented and this combination enhanced the expression of an antigen-specific IgG response without the induction of IgE [62].

### 3.2. AIT Adjuvants in Prelinical Development

#### 3.2.1. CpG Oligodeoxynucleotide

CpG oligodeoxynucleotides (ODNs) are synthetic, non-methylated DNA molecules that code for cytosine and guanine triphosphate deoxynucleotide base pairs [63]. CpG motifs conjugated with allergen, facilitate the uptake of allergen and activate TLR9 (toll like receptor 9) in the endosome, leading to the differentiation of naive T cells to Th1, further leading to the production of Th1-promoting cytokines like IFN-γ. This would restrain Th2 immune responses and IgE antibody production, thereby, reducing allergic symptoms like asthma and inflammation [64,65]. CpG ODN being linked directly onto the allergen has displayed great promise as a therapeutic and prophylactic system for allergic diseases in preclinical studies [66]. Moreover, delivering CpG ODN on nanostructures that have the potential to serve as platforms for several allergens have performed similarly well in preclinical and even clinical studies [67,68].

#### 3.2.2. Vitamin D3

Vitamin D exhibits the ability to induce Treg cells and thus could serve as an effective adjuvant for AIT. Vitamin D acts by inhibiting the maturation of dendritic cells, enhancing IL-10 secretion, and upregulation of Foxp3-positive CD4-positive T cell [69]. Petrarca et al. investigated the effect of vitamin D3-adjuvanted allergoid vaccine for house dust mite allergy with a low dose of allergen, and found a prominent reduction of airway eosinophilia and Th2 cytokines in a Der p 2-sensitised BALB/c mice model. A concomitant increase of Treg cells and IL-10 in the lung and Der p 2-specific IgG2a in the serum were also observed. This study indicated an effective, economic AIT strategy for house dust mite allergy [70]. Furthermore, recombinant Fel d 1 (cat allergen) coupled to vitamin D3 exhibited beneficial effects by reducing airway inflammation, airway hyper-responsiveness, and initiation of allergen-specific immune responses [71]. Although these studies open a novel platform for vitamin D3 as a safe and effective adjuvant, further validation on the adjuvant action of vitamin D3 are pertinent.

The merits, demerits, and the present status of each adjuvant in AIT are detailed in Table 1.

## 4. Potentials of Nanomedical Platforms

Immunotherapies for cancer and infectious diseases utilize nanotechnology to enhance the efficacy and safety of treatment [72,73]. Similar bionanomedical approaches have been proposed for the development of more effective adjuvants in AIT, circumventing the negative side effects. Concern has been raised for classical adjuvants being able to induce autoimmune reactions, summarized as autoimmune syndrome induced by adjuvants (ASIA)/Shoenfeld’s Syndrome [74,75]. While there is a clear definition of nanomaterials as having a size lower than 100 nm, in nanomedicine, carrier systems often exceed this strict confinement. A comprehensive and thorough evaluation of safety of the constituents in a medical formulation represents the integral part of pharmaceutical development [76]. Nanomedicine displays numerous potential in pharmaceutical development. Thus, nanomedical platforms need to be subjected to the same precise assessment as that of other pharmaceuticals. There have been numerous studies investigating the safety aspects of nanomaterials, which includes toxicity assessment, identification of potential medical hazards, and risk assessments [77]. In particular, positively charged and hydrophobic surface characteristics of nanomaterials have raised safety concerns, however, for AIT surface modification using specific ligands are intended for the improvement of this treatment, as will be discussed below. Some of the specific salient properties of nanoparticles, such as defined size, ease of production, functionalization, targeting ability, and suitability to be engineered in a tailored way, based on the type of allergen, make them safer and potentially more efficacious candidates for novel vaccines in AIT. Selected properties of nanomedical platforms that might render them attractive are discussed in the following section.

### 4.1. Physicochemical Properties of Nanoparticles

Some of the physicochemical properties exhibited by nanomaterials can directly or indirectly aid in immunomodulation. The small size of nanoparticles can improve their tissue permeation and thereby enhance the availability of particle-loaded antigen to the blood vessels and lymph nodes. Palmer et al. studied the effect of transdermal delivery of amorphous silicon dioxide nanoparticles in a contact dermatitis allergy model, and reported their enhanced immunomodulatory potential [78]. This study compared the skin permeability of nano- vs. microparticles, showing improved penetration of nanoparticles (27.8 +/− 3.4 nm) compared to microparticles (557.6 +/− 35.1 nm) in the skin of mouse [78]. Similarly, Hirai et al. investigated the skin permeation and subcellular localization of monodisperse amorphous silica nanoparticles sized 70 nm, in mice, and demonstrated that the particles penetrated effectively through the skin barrier and were localized in the lymph nodes [79]. There are ample studies demonstrating the efficacy of nanomaterials to penetrate skin barriers [80,81]. Effective permeation and localization in the tissues can in fact improve AIT through novel routes of administration like epicutaneous or intranasal delivery. In addition to the size, the surface charge, chemical composition, shape and solubility makes them attractive for AIT. Jatana et al. determined the influence of size and charge of different nanoparticles (gold, silver, silica, and titanium dioxide) on immunomodulation in a mouse model of allergic contact dermatitis and concluded that small and negatively charged nanoparticles exhibited immunosuppressive effects [82]. Biodegradable polymeric nanoparticles that are biocompatible with tissues and cells, can be promising candidates in AIT that might reduce the unwanted side effects associated with the current therapy, using alum as adjuvant. The shape and surface properties of nanoparticles have a huge impact on the cellular uptake. A study published by Champion et al. demonstrated that one could possibly control phagocytic uptake through efficient shape design. They found that spherical particles are taken up more effectively by macrophages due to their high length-normalized curvature (the length over which a curvature exists) [83]. The surface characteristics of the particle can indeed promote uptake by inducing interactions with cell surface receptors. Therefore, this property can be exploited through surface functionalization of nanoparticles with the desired functional groups binding the specific surface receptors. Lectin-functionalized polylactic-co-glycolic acid (PLGA) particles were proposed as a promising platform for oral AIT. This functionalization approach was efficient in targeting enterocytes, thereby improving uptake and preventing degradation of the delivered antigen through gastrointestinal enzymes [84].

### 4.2. Ability to Form a Depot

The depot effect is still considered as one of the important mechanisms for induction of immune tolerance. It has been recognized that the persistence and prolonged release of allergen can increase the immune cell’s exposure time and can lead to immunomodulation [85]. Moreover, formation of a depot at the target site concomitantly reduces the dose of therapy. In the case of nanoparticles, the antigen or allergen can be encapsulated inside the carrier system and its release can be tuned to function at a desired level by modifying the particle surface with polymers. Chitosan coating of drug-encapsulating PLGA nanoparticles exhibited a controlled release of active pharmaceutical ingredients, compared to pristine PLGA nanoparticles [86]. Lacey et al. demonstrated an immune enhancing effect of the depot formation of cationic liposome-containing tuberculosis vaccine antigen (Ag85B–ESAT-6) [87]. Thus, the ability of nanoparticles to form a depot could have therapeutic benefits.

### 4.3. Protection from Enzymatic Degradation

Nanoparticles can protect the encapsulated antigen by shielding it from the proteolytic enzymes in the body, which is especially desired during oral immunotherapy (OIT) as the vaccine has to surpass the harsh conditions of the gastrointestinal tract [88]. OIT represents a novel strategy under investigation for the treatment of food allergies. It has been shown to be effective in about 60 to 80 percent of the population studied [89]. Brotons-Canto et al. investigated the ability of mannosylated nanoparticles for OIT against peanut allergy in mice and concluded a high suitability for AIT. The nanoparticle system was synthesized from a novel polymer obtained by the covalent binding of mannosamine with a polyanhydride backbone [90]. Similarly, oral immunotherapy with polyanhydride nanoparticles was reported to have potential benefits in the treatment of peanut allergy. The study exhibited a surge in Th1 and Treg immune response and a declined Th2 cell activation in in vivo mouse models. Furthermore, Srivasta et al. demonstrated success of a preclinical study of OIT using CpG-coated PLGA nanoparticles in murine models for peanut allergy [91]. They observed a sustained and significant decrease in peanut-specific IgE/IgG1 levels, together with Th2 cytokines and an increase in peanut-specific IgG2a levels and IFN-γ. The study also detailed the safety of CpG-PLGA NPs in OIT by stating their inability to induce anaphylactic symptoms, by measuring the plasma histamine release [91]. Thus, the aptness of nanoparticles in protecting the allergen along with the activation of desired immune response makes them ideal for AIT.

### 4.4. Enhancement of Allergen-Specific Tolerance

The ultimate goal of immunotherapy in allergic disorders is to induce allergen-specific immune tolerance. The inherent capability of nanoparticles to target the APCs and the ability to transmit signals that evoke an antigen-specific immune response makes them an implementable tool to modulate immune responses [92]. Those nanoparticles that can achieve this purpose of immune tolerance induction can be collectively termed as tolerogenic nanoparticles. Application of tolerogenic nanoparticles for AIT would be appropriate in preventing unwanted immune reactions that tend to occur in the current treatment practices. A diverse set of nanomaterials were applied in inducing tolerogenic immune responses. Metals or metal oxide nanoparticles, liposomes, synthetic polymers are being studied, but biodegradable polymeric nanoparticles are the most popularly investigated nanomaterial in this aspect [93,94]. Maldondo et al. reported the potential of a tolerogenic nanocarrier system (encapsulated with protein/peptide antigen) to prevent and neutralize a pathological immune response [95]. Moreover, polymeric (pluronic-stabilized polypropylene sulfide) nanoparticles conjugated with CpG ODNs were proven to be functional in the treatment of house dust mite-induced allergic airway disease [96]. The pulmonary administration of nanoparticle conjugated with CpG showed a pronounced enhancement in dendritic cell (DC) recruitment and activation, resulting in a Th1 immune response in a house dust mite allergy model and reduced allergy-associated symptoms. These studies indeed recommend further expansion of nanomaterial as a potential platform technology for the mitigation of allergic diseases.

## 5. Harnessing the Adjuvant Effect of Nanoparticles

**Figure 3:**
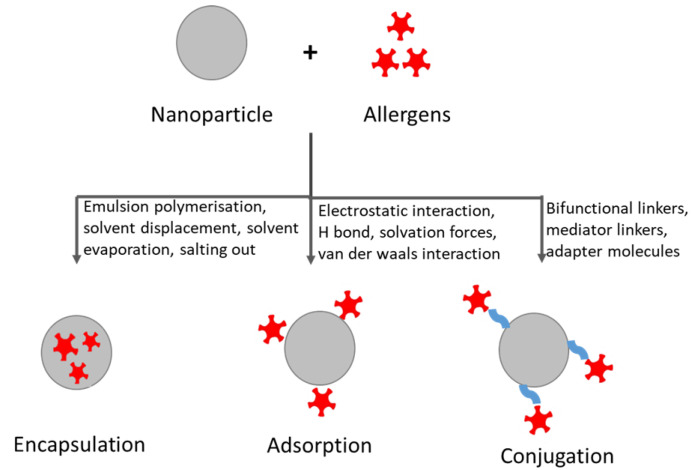
Methods of loading allergens into or onto nanoparticles.

The design of AIT vaccine with nanoparticles involves the loading of the desired allergen onto their surface either through physical adsorption, through direct covalent conjugation, or through nanoparticle encapsulation (Figure 3) [97]. The process of encapsulation of an allergen is highly advantageous in mucosal or oral AIT. These two routes of AIT are highly susceptible to enzymatic degradation and, thus, the encapsulation process can pose advantages as it shields the allergen from catabolic degradation. Additionally, targeting specific cells or tissues can be achieved, which would further result in delivering allergen at an optimal dose and in minimizing adverse effects [98]. Physical adsorption, in contrast, can be considered to be a relatively simple method where the least amount of stress is applied on the allergens, putatively causing minimal damage of allergens (i.e., structural alterations, epitope rearrangement) [99]. Of note, while covalent conjugation techniques result in the formation of a stable allergen–nanoparticle complex, undesirable structural alterations of allergen might occur, leading to steric hindrance. To counteract this issue, different spacer molecules of optimal length and chemistry can be used [100].

The properties of nanoparticles such as the small size, propensity for surface modification, biodegradability, biocompatibility, and simple synthesis protocols make them ideal prospective adjuvants for AIT. Many organic and inorganic nanoparticles have been studied for their immunological properties, also particularly in regard to their ability to induce suppression of detrimental immune responses in allergy [101]. Tahara et al. evaluated the effect of PLGA nanoparticles on the degranulation of mast cells following antigen exposure in a mouse model for systemic anaphylaxis and found reduced antigen-induced allergic responses [102]. Over the last years, several nanoparticles-based delivery systems have been tested, revealing the adjuvant potential of in vivo models [103]. Biodegradable PLGA nanoparticles encapsulated with allergen led to the generation of IgG antibodies and Th1 cytokine milieu in a mouse model for allergic asthma, suggesting an efficient Th1 response [104]. However, there are still gaps in understanding the immune response generated by nanoparticles loaded with allergen. Thus, it is necessary to study the influence of nanoparticle–allergen complexes on mechanisms that regulate immune responses, such as allergen processing and presentation. Here, we discuss the major mechanisms influenced by the allergen when associated with nanoparticle on immune modulation.

### 5.1. Recognition and Internalisation by APCs

Antigen presenting cells play a critical role in the activation of the innate and adaptive immune system. The foremost step in the activation of the immune system involves antigen recognition and internalization. While the allergens themselves are not recognized by the immune cells using pattern recognition receptors (PRRs), there might be recognition associated with allergen exposure if the allergen carrier (e.g., plant pollen, animal hair, dust mite dander) has also associated other materials such as lipopolysaccharides, which are recognized by PRRs, including C-type lectin receptor (CLR) and TLRs. In case of nanoparticles, their physicochemical properties, such as size, surface chemistry, and shape govern their cellular internalization, leading to significant differences in their mechanism of uptake [105]. In general, nanoparticles are taken up by immune cells via three pathways—phagocytosis, micropinocytosis, and receptor-mediated endocytosis. Polypropylene nanoparticles, for instance, were found to be internalized by receptor-mediated endocytosis [106]. Similar endocytosis mechanisms into dendritic cells were observed in chitosan-coated PLGA nanoparticles of 150 nm, whereas gold nanoparticles of 30–50 nm were phagocytosed [107,108]. An enhanced uptake of nanoparticle-associated antigen was reported in APCs. Uto et al. reported an increased efficiency in the uptake of biodegradable poly-γ-glutamic acid nanoparticles of 250 nm functionalized with ovalbumin [109]. An increased efficiency of nanoparticle-mediated update of antigens should help to accomplish the same or an even better efficacy compared to conventional AIT, even with a lower dose of antigen. Thus, AIT with nanoparticles can become more effective with less adverse effects. However, there are to our knowledge no studies reporting the influence of nanoparticle-associated allergen on antigen uptake by APCs. Hence, a detailed and comprehensive research into the mechanisms and kinetics of nanoparticle-mediated allergen internalization are mandatory.

### 5.2. Maturation of APCs

Maturation of APCs plays a critical role in the efficient priming of naive T cells for an efficient T cell response in AIT [110]. Initially, APCs exist in their immature state characterized by the expression of surface receptors (phagocytic or scavenging), which include CD91, integrins, CD36, and pattern recognition receptors (TLRs, NLRs). Pattern recognition receptors assist in the recognition of signals like pathogen-associated molecular patterns (PAMPs) and damage-associated molecular patterns (DAMPs) by APCs [111]. During the conversion of APCs from immature to mature state, modifications occurs in both phenotypical and functional levels, such as downregulation in the expression of surface endocytic receptors, along with an upregulation of co-stimulatory molecules and chemokine receptors [112,113]. Phenotypical maturation is characterized by the expression of CD80, CD83, CD86, and major histocompatibility complex class II (MHC class II), whereas the functional maturation is measured by the balance in the level of secretion of both pro-inflammatory and anti-inflammatory cytokines [114,115]. The maturation of APCs can be categorized into a mature and semi-mature states based on the strength and persistence of the exogenous stimulatory signals. Development of allergic disease is characterized by the overexpression of surface markers such as CD80 and CD86, and increased expression of specific pro-inflammatory mediators (IL-4, IL-5) [116]. Nanoparticles have been documented to both promote and to inhibit maturation of APCs, based on their physicochemical properties and concentration. Shima et al. observed the impact of amphiphilic poly (γ-glutamic acid) nanoparticles with a size range of 30–200 nm on the maturation of DCs in mouse models. They showed that the small size of nanoparticles combined with their large surface area resulted in highest maturation, measured by the expression of CD80, CD86, which might be attributed to their efficient interaction with the DCs [117]. ZnO nanoparticles upregulated the expression of co-stimulatory molecules CD80 and CD86, and the secretion of IL-6 and TNF-α, at a concentration of 30 μg/mL, but no such effects were observed at lower concentrations (10 μg/mL) [118]. Gold nanoparticles of 10 nm inhibited the expression of CD86, CD83, MHC II, and IL-12p70 induced by LPS treatment in DCs [119]. It is hypothesized that immune cells can recognize nanoparticles due to the presence of surface-adsorbed biomolecules, or recognize specific structures of the nanomaterial, which are designated as nanoparticle-associated molecular patterns (NAMPs) [120,121]. Similar to pristine nanoparticles, nanoparticle-antigen conjugates are shown to enhance the maturation of APCs. Chitosan nanoparticles of 254 nm encapsulating the model antigen ovalbumin stimulated an increased expression of surface maturation markers, such as CD40, CD80, CD86, MHC class I, and II, along with secretion of pro-inflammatory cytokines IL-1β, IL-6, IL-12p70, and TNF-α [122]. Additionally, surface functionalization of ovalbumin to 210 nm poly (γ-glutamic acid) nanoparticles amplified DC maturation, when compared to the native ovalbumin [109].

The deviation of allergen-specific effector T cells from Th2 to a regulatory phenotype (peripheral tolerance) is considered to be a successful outcome in AIT [123]. Peripheral tolerance can be induced by semi-mature DCs marked by an increased expression of the co-stimulatory molecules CD80 and CD86, as well as by release of the anti-inflammatory cytokines IL-10 and transforming growth factor-β (TGF-β) [111,124,125]. Moreover, a recent study applying the conjugation of TGF-β to antigen-loaded nanoparticles has shown promise in strengthening the regulation of antigen-specific tolerance [126]. In summary, a number of approaches based on the modulation of APC maturation can result in the development of immune tolerance.

### 5.3. Antigen Processing and Presentation

During antigen processing, endocytosed proteins are gradually degraded into peptide fragments. This process involves the endolysosomal compartment of APCs, where a suite of proteolytic enzymes degrades proteins into peptides, under the influence of an increasingly acidic pH [127]. The resulting peptide fragments are then displayed on the surface of the APCs in association with MHC class II molecules, for recognition by CD4+ T lymphocytes [128]. These processes together result in T cell activation. Further, the activated T cells are stimulated by co-stimulatory molecules expressed on the surface of APCs, which interact with CD28 or cytotoxic T lymphocyte-associated protein 4 (CTLA-4) [129]. Allergens exhibit different processing kinetics along with variable peptide fragmentation, even if they share common structural features with non-allergenic proteins [130]. Allergenicity of an antigen can be characterized by its limited susceptibility for endolysosomal processing. Mutschlechner et al. investigated the processing and presentation of Bet v 1 (major birch pollen allergen) in dendritic cells and concluded that its high allergenic potential was attributed due to its increased resistance for proteolytic degradation (processing) [131,132]. Nanoparticles have been found to influence antigen processing. For instance, polyvinyl alcohol-coated super-paramagnetic iron oxide nanoparticles (PVA-SPIONs) showed a significant decrease in antigen processing and presentation by monocyte-derived dendritic cells (MoDC) using albumin as a model antigen [133]. To investigate this, DCs were treated with PVA-SPIONs and the uptake was analyzed by flow cytometry (FACS), while their activation, function, and stimulatory capacity were assessed by FACS and in vitro CD4+ T cell assay. It was found that the particles were taken up by actin-dependent mechanisms and showed a decrease in antigen processing and MHC class II molecules expression. A reduced T lymphocyte activation and an increased release of anti-inflammatory cytokines (mainly IL-10) were also observed, which revealed that DCs are reverted to an immature or semi-mature state, which favored the induction of peripheral tolerance [133]. Furthermore, graphene oxide nanoparticles were associated with ovalbumin-impaired antigen processing and presentation in bone marrow-derived DCs [130]. The planar and negatively charged surface of graphene oxide nanoparticles was considered to be the contributing factor [134]. Single-walled carbon nanotubes compromised the antigen capture/processing and presentation function of DCs, without affecting their maturation process [135].

Compromised antigen processing might indeed suggest decreased allergenicity and the induction of a tolerant state in peripheral T cells (IL-10-dependent immunologic tolerance). This phenomenon can be harnessed in the development of vaccines for allergy. As a potential adjuvant, nanoparticles could lead to the generation of Treg cells and cause a suppression of Th1- and Th2-specific allergen responses. However, further investigations are necessary to extend our understanding about this aspect.

### 5.4. T Cell Differentiation

Presentation of antigen to the naive T cells along with the costimulatory molecules and cytokines eventually leads to the proliferation and differentiation of naive T cells into specific effector T cell subsets [136]. Among all factors, cytokine environment plays a dominant role in cell differentiation. Nanoparticles impact T cell differentiation. Thermally hydrocarbonized, oxidized porous silicon nanoparticles (TOPSi, THCPSi) enhanced T cell proliferation, assayed by a co-culture of peripheral blood lymphocytes and MoDCs [137]. Magnetic iron oxide and PLGA nanoparticles were observed to induce Th1 proliferation [138,139]. In addition to this, tolerogenic nanoparticles have been reported to induce antigen-specific Treg cells [140]. However, impairment in the activation capacity was observed in PVA-SPIONs when CD4+ T cells were co-cultured with MoDCs [134]. Differences in the properties of nanoparticles, along with variations in nanoparticles composition and treatment conditions can lead to modulation of the T cell response [141].

**Figure 4:**
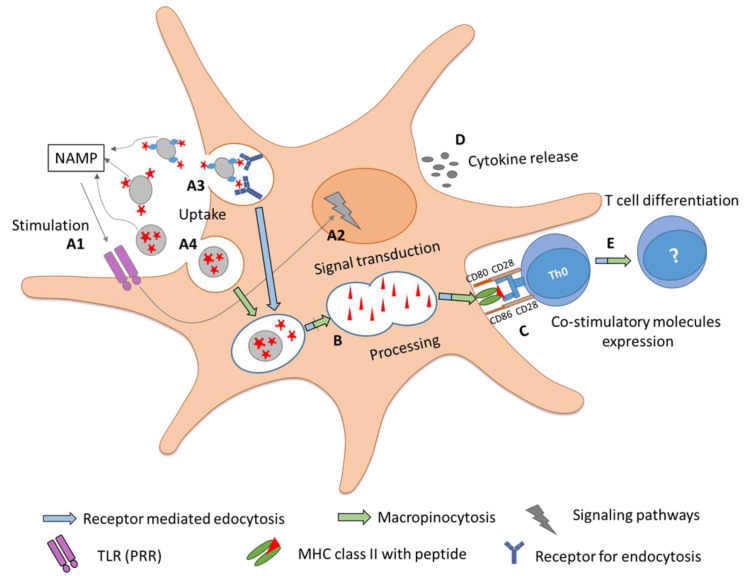
Adjuvanticity of nanoparticles along with the events associated in developing immune tolerance. In event A1, stimulation of immune responses by nanoparticles can be associated with NAMPs that can induce (event A2) signaling cascades, leading to the transcription of maturation genes. Nanoparticles can increase the uptake of allergen mainly by (event A3) macropinocytosis or (event A4) receptor-mediated endocytosis, based on the physicochemical properties of nanomaterial. In event B, nanoparticles associated with allergens can increase the proteolytic processing and lead to maturation of APCs marked by enhanced (event C) co-stimulatory molecule expression and (event D) cytokine release (IL-10, TGF-β), which further triggers (event E) T cell activation and differentiation.

Although a few molecular mechanisms determining the influence of nanoparticle allergen complexes have been investigated, the majority still have to be revealed. A detailed investigation of these processes can provide a better insight into the adjuvant effects of nanoparticles for AIT. Figure 4 depicts the possible roles of nanoparticles in AIT.

## 6. Future Perspectives

A prospective vaccine for AIT should be able to accomplish (a) safety and efficacy with highest possible dose of allergen, (b) patient compliance that is promoted by a well-tolerable regimen, cost effectiveness, self-administration and (c) minimization of adverse effects associated with the therapy. Nanoparticles can in principle fulfil the above-mentioned criteria and their physiochemical properties and an inherent ability to induce immune tolerance might qualify them as an ideal type of adjuvant for AIT. Even though in vivo studies with nanoparticles hold promising results, a deeper understanding of nanoparticle–allergen complexes and their interaction with the immune system at a molecular level is mandatory. A safe and efficient adjuvant for AIT can be attained by exploring the influence of the allergen–nanoparticle complex on each mechanistic step underlying the immune response, such as uptake, maturation, antigen processing, presentation, and induction of T cell differentiation.

## Figures and Tables

**Table 1:** Adjuvants for AIT with their proposed mechanism of action, merits, demerits, and status in the market.

Adjuvant	Proposed Mechanism of Action	Merits	Demerits	Status
Alum	Depot effect NLRP3 inflammasome activation Induction of self-DNA release	Wide applicability in vaccines	Adverse effects Induction of autoimmune or Th2-based immune responses Non-biodegradable Gaps in safety and toxicity data	On the market for AIT
Calcium phosphate	Depot effect	Biodegradable and biocompatible	Local adverse reactions Lower adjuvant activity compared to alum.	On the market for AIT
Microcrystalline tyrosine	Depot effect	Biodegradable and biocompatible Good local and systemic tolerance	Not suitable for patients with tyrosine metabolic disorders.	On the market for AIT
Monophosphoryl lipid A	TLR4 agonist APC activation Immune cascade induction	Stronger and long-lasting immune response Reactogenic at the site of injection	Production variability with different batches. Low bioavailability by itself	On the market for AIT
CpG oligonucleotide	TLR9 agonist APC activation Immune deviation	Co-administration with other adjuvants can overcome Th2 bias Strong Th1 immune response	Degradation by DNase. Short half life Decreased uptake due to the negative charge Reduction of antigen dose	Clinical trial phase

## List of Abbreviations

1. APCs: Antigen presenting cells
2. CBPs: Carbohydrate particles
3. CD 86, 83: Cluster of differentiation 86, 83
4. CLR C: type lectin receptor
5. CTLA-4: Cytotoxic T lymphocyte-associated protein 4
6. DAMPs: Damage-associated molecular patterns
7. DC: Dendritic cells
8. DNA: Deoxyribonucleic acid
9. FACS: Fluorescence-activated cell sorting
10. Foxp3: Forkhead box P3
11. IFN-γ: Interferon-γ
12. IgE: Immunoglobulin E
13. IgG: Immunoglobulin G
14. IL-10: Interleukin 10
15. LPS: Lipopolysaccharides
16. MAT: Modular antigen transporter
17. MCT: Microcrystalline tyrosine
18. MHC: Major histocompatibility complex
19. MPL: Monophosphoryl lipid A
20. NAMPs: Nanoparticle-associated molecular patterns
21. NLRP3: NOD-like receptor protein 3
22. ODNs: Oligodeoxynucleotides
23. OIT: Oral immunotherapy
24. PAMPs: Pathogen-associated molecular patterns
25. PLGA: Polylactic-co-glycolic acid
26. PRRs: Pattern recognition receptors
27. TGF-β: Transforming growth factor-β
28. Th1, Th2: T helper cells 1, 2
29. TLR9: Toll-like receptor 9
30. TLRs: Toll like receptors
31. Treg: Regulatory T cells
32. TRIF: TIR-domain-containing adapter-inducing interferon-β
